# Single-Walled Carbon Nanohorns for Energy Applications

**DOI:** 10.3390/nano5041732

**Published:** 2015-10-21

**Authors:** Zhichao Zhang, Shuang Han, Chao Wang, Jianping Li, Guobao Xu

**Affiliations:** 1 College of Applied Chemistry, Shenyang University of Chemical Technology, Shenyang 110142, China; E-Mails: unizhang@syuct.edu.cn (Z.Z.); unihanshuang@syuct.edu.cn (S.H.); 2 College of Chemistry and Bioengineering, Guilin University of Technology, Guilin 541004, China; E-Mail: chaowang@ciac.ac.cn; 3 State Key Laboratory of Electroanalytical Chemistry, Changchun Institute of Applied Chemistry, Chinese Academy of Sciences, Changchun 130022, China

**Keywords:** single-walled carbon nanohorn, fuel cell, solar cell, biofuel cell, Li-ion batteries, supercapacitor, hydrogen storage

## Abstract

With the growth of the global economy and population, the demand for energy is increasing sharply. The development of environmentally a benign and reliable energy supply is very important and urgent. Single-walled carbon nanohorns (SWCNHs), which have a horn-shaped tip at the top of single-walled nanotube, have emerged as exceptionally promising nanomaterials due to their unique physical and chemical properties since 1999. The high purity and thermal stability, combined with microporosity and mesoporosity, high surface area, internal pore accessibility, and multiform functionalization make SWCNHs promising candidates in many applications, such as environment restoration, gas storage, catalyst support or catalyst, electrochemical biosensors, drug carrier systems, magnetic resonance analysis and so on. The aim of this review is to provide a comprehensive overview of SWCNHs in energy applications, including energy conversion and storage. The commonly adopted method to access SWCNHs, their structural modifications, and their basic properties are included, and the emphasis is on their application in different devices such as fuel cells, dye-sensitized solar cells, supercapacitors, Li-ion batteries, Li-S batteries, hydrogen storage, biofuel cells and so forth. Finally, a perspective on SWCNHs’ application in energy is presented.

## 1. Introduction

The industrial revolution in the mid-eighteenth century offered people abilities far beyond animal and human power. The internal combustion engine, and later electricity and related technologies, are all based on consumption of fossil fuels. Increasing demand for energy comes from worldwide economic growth and development. The global total primary energy supply (TPES) mainly relies on fossil fuels. Among the many human activities that produce greenhouse gases, the combustion of fossil fuels represents by far the largest source of CO_2_ emissions, which severely threaten the world’s security, for instance, via global warming. In 2012, global CO_2_ emissions were 31.7 Gt (gigatoes (Gt), tonne of oil equivalents (t.o.e.)) [1,2]. As the world revolution continues, reliable energy supply is of great importance and urgency. In 2013, world TPES was 13,555 million tonnes of oil equivalent (Mtoe) of which 13.5%, or 1829 Mtoe was produced from renewable energy sources. Though renewable energy resources have emerged, the majority of energy consumption nowadays is still carbon-based. Thus, high energy efficiency, devices for energy storage, sustainable clean energy sources and protection of our environment are vital to our society. For instance, utilization of sunlight which can be converted to electricity and stored in batteries or capacitors is one of the most popular methods to obtain sustainable clean energy. However, many scientific issues remain unsolved and a significant breakthrough is still needed to provide practical end-use for these technologies. Hence, one active research direction is to develop new materials for energy applications. This includes high-performance materials with specific characteristics, for example as electrode materials for lithium-ion batteries, supercapacitors, fuel cells, and host materials for hydrogen storage. Nanocarbon materials play an important role from this perspective.

Recently, a new nanocarbon material, namely single-walled carbon nanohorns (SWCNHs), has become the focus of intense research owing to its unique physico-chemical properties and versatile applicability [3]. SWCNHs are a kind of nanocarbon of high purity since no metal catalyst is involved in the process of synthesizing them by laser ablation on highly purified graphite. They are structures of single-graphene tubules with highly strained conical ends. Generally, sp^2^-hybridized carbon exhibits a high diversity in crystallinity, morphology, porosity, and texture. The chemical, physical, and electronic properties of SWCNHs can be finely tuned by structural manipulation, which is vital to the design of high-end electrochemical devices. Their surface area can be easily modified by a variety of methods including heat treatment in oxidative gases and acid treatment, which may significantly influence the final application. Thus, the surface area could be greatly enhanced, to as high as 1000 m^2^/g as a result of horn-opening. Meanwhile, the internal tubular area becomes available for reactants as well. Moreover, the chemical defects and crystal edges of SWCNHs confer good electrocatalytic properties and make them good candidates for iodide/triiodide redox reactions. The processability of SWCNHs in solution not only affords films with high porosity and a large surface area, but also produces hybrid materials when other materials are employed, displaying superior properties over any single components. Thus, SWCNHs have attracted a great deal of attention for various potential applications, such as electroanalysis [4,5,6,7,8], electrochemiluminescence [9], biosensors [10,11,12,13,14], fluorescent detection [15,16,17], colorimetric detection [18], gas storage [19], catalyst supports [20], drug carrier systems [21], and optoelectronic devices in recent years [22,23]. In this review the highlights will be focused on the fabrication of SWCNH-based nanohybrids or nanocomposites and their energy applications, such as fuel cells, solar cells, supercapacitors, and lithium ion batteries.

## 2. Characteristics of SWCNHs for Energy Applications

A number of characteristics of SWCNHs render them attractive materials for energy applications, such as high surface area, tunable pore structure and high electron, phonon and heat transport. They are usually *ca.* 2–5 nm in diameter and *ca.* 40 to 50 nm in length, and self-assemble to form dahlia-like spherical aggregates with diameters ranging from 80 to 100 nm, rendering large amount of surface area (*ca.* 300 m^2^/g) while maintaining high electrical conductivity for close contact among SWCNHs [3,24]. As-grown SWCNHs are closed, but holes can be opened in their walls easily by treating them in an oxygen atmosphere or acid. The size and number of holes can be controlled by adjusting the hole-opening conditions. Due to their small size and the specific morphology, the reactant/product in a chemical reaction or the charged particles (ions or protons) can be better transported to the active sites of SWCNHs [22]. Their detailed characteristics have been reviewed previously [21].

## 3. SWCNHs for Energy Conversion

SWCNHs are finding increasing applications in the highly challenging area of energy conversion systems, from the traditional area of fuel cells to the development of advanced solar cells as well as new areas of development such as biofuel cells and solar thermal collectors. SWCNHs are novel candidates for catalyst support or even catalysts. As catalyst support, scientists often load catalyst nanoparticles on SWCNHs to form hybrid materials. The application requires uniform nanoparticles attached to the SWCNHs surface to provide a high active specific surface area. The unique structure of SWCNHs would improve the durability of catalysts [25]. In addition, research finds that heteroatom doped SWCNHs have a certain catalytic activity. The development of non-precious metal catalysts is an emerging area of research aimed at providing a cost-effective alternative to the traditional energy market [26].

### 3.1. SWCNHs for Fuel Cell

#### 3.1.1. Deposition of Single Metal on SWCNHs

A number of methods for deposition of Pt catalyst on SWCNHs used in fuel cells have been reported—for example, by using a colloidal method or by arc plasma in liquid nitrogen using a Pt-contained graphite anode [27,28]. The Pt nanoparticles less than 5 nm were homogeneously dispersed on the SWCNHs.

**Figure 1:**
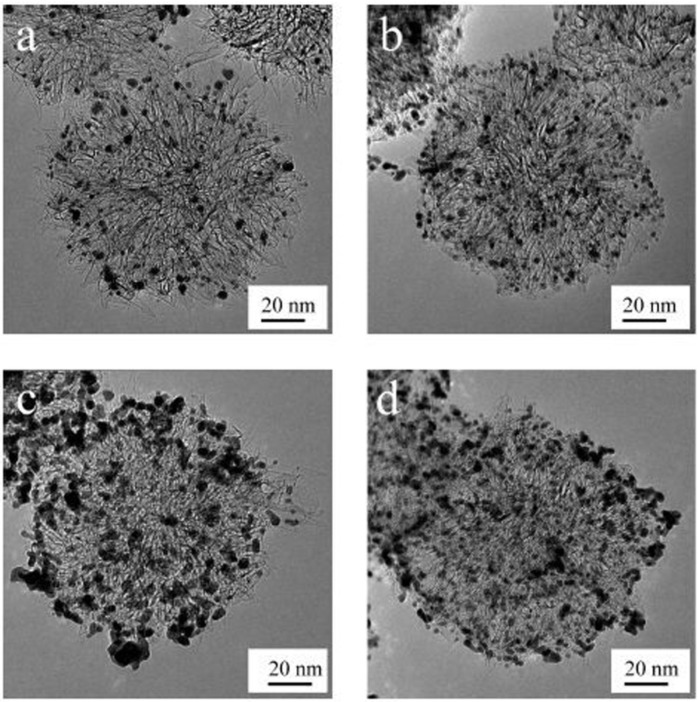
Transmission electron microscope (TEM) images of the supported catalyst: (**a**) 20 wt % Pt on as-grown single-walled carbon nanohorns (SWCNHs); (**b**) 20 wt % Pt on treated-SWCNHs; (**c**) 60 wt % Pt on as-grown SWCNHs; (**d**) 60 wt % Pt on treated SWCNHs. Reproduced with permission of [29]; American Chemical Society, 2009.

Open-SWCNHs have a high nanoporosity of different interaction potential for molecules. The interstitial pores and nanowindows can offer the stable sites for nanoparticles on the SWCNH samples. Kubo’s group intentionally created defects on the surface of SWCNHs by oxidizing them with H_2_O_2_ in order to prevent the growth of Pt nanoparticles even under high-Pt-content conditions [29]. 2.9 nm Pt nanoparticles were highly dispersed on oxidized SWCNH samples (ox-SWCNHs), which was roughly 2/3 that of the Pt particles on as-grown SWCNHs, for 60 wt % Pt content. They obtained a high-power direct methanol fuel cell at 40 °C with a power density of 76 mW·cm^−2^ at 0.4 V (Figure 1).

Moreover, uniform and well-dispersed Pt nanoparticles were successfully deposited on as-grown SWCNHs and ox-SWCNHs when using 4,4′-bipydine and ion liquids as link agents, respectively [30]. The size of Pt nanoparticles were controlled in a very narrow range (2.2 to 2.5 nm) when ion liquids were applied. Ox-SWCNHs were better than as-grown SWCNHs in obtaining Pt nanoparticles with good dispersibilty and uniform size. The obtained nanocomposites had much better electrocatalytic activity for the methanol oxidation than those prepared with carbon nanotubes as supporter.

In another report, “unprotected” Pt nanoclusters were deposited on nitrogen-doped SWCNHs (N-SWCNHs) to form a highly durable and active nanocomposite cathode catalysts (Pt/N-SWCNHs) [31]. The specific catalytic activity and mass catalytic activity for the oxygen reduction reaction over Pt/N-SWCNHs were much better than a commercial Pt/C catalyst (Pt/C-JM). There was no obvious loss in the catalytic activity of Pt/N-SWCNH after potential cycling from 0.6 to 1.1 V *versus* RHE for 15,000 cycles at 30 °C, under the oxidizing conditions for the electrochemically catalytic reduction of O_2_. During the accelerated aging tests, Pt nanoparticles in Pt/N-SWCNH were more stable than those in Pt/C-JM, showing a low increase in the particle size.

The performance of electrodes prepared with electrocatalysts based on Pt overloaded SWCNHs (Pt-SWCNHs) was also compared with that in carbon black (Pt-carbon black) for high temperature fuel cells [32]. The ohmic resistance for the Pt-SWCNHs was higher than the carbon black-based membrane electrode assemblies, due to the higher hydrophobic character of the SWCNHs support. Furthermore, the Pt-SWCNHs anode presented a lower charge transfer resistance than the corresponding carbon black with similar cathode charge transfer resistance. The authors also studied the polymer electrolyte membrane fuel cell (PEMFC) performance when using as-prepared and oxidized SWCNHs to support Pt nanoparticles [33]. Two different oxidizing treatments were considered: oxygen flow at 500 °C and reflux in an acid solution at 85 °C. Oxygen treatment increased surface area 4 times while acid treatment increased 2.6 times. Acid treatment of SWCNHs increased chemical fragility and decreased electrocatalyst load in comparison with as-prepared SWCNHs. While the oxygen-treated SWCNH sample allowed us to obtain the highest electrocatalyst load, the use of as-prepared and oxygen treated SWCNHs showed, in both cases, catalytic activities 60% higher than using conventional carbon black as electrocatalyst support in polymer electrolyte membrane fuel cells. Moreover, electrochemical impedance spectroscopy analysis indicated that the major improvement in performance is related to the cathode kinetics in the as-prepared SWCNHs sample, while concerning the oxidized SWCNHs sample, the improvements are related to the electrokinetics in both anode and cathode electrodes. These improvements should be related with differences in the hydrophobic character between SWCNH and carbon black.

Mohamedi’s group discussed the effect of the carbon morphology in binderless nanostructured Pt catalyst on the oxygen reduction reaction [34]. It seems that the dimensions (diameter and length) are irrelevant since the three carbons (carbon nanofibers (CNF), carbon nanotubes (CNT) and SWCNH) displayed similar supporting properties regarding either the onset potential or the half-wave potential of oxygen reduction reaction.

Besides exploiting methods of loading Pt on SWCNHs, scientists have developed new synthetic methods of SWCNHs to get higher specific surface area and better dispersivity of Pt nanoparticles on them. SWCNHs were synthesized by a gas-injected arc-in-water method at low cost. The as-grown SWCNHs possessed a high specific surface area when the arc discharge current was optimized with continuous arc mode. Such a product can support Pt catalyst with high dispersivity, leading to high performance in PEFCs as catalyst layers [35,36].

The first direct laser based synthesis of SWCNHs onto carbon microfibers for the straightforward fabrication of free-standing (binderless) electrodes has been reported [37]. These SWCNHs have diameters as small as 2–4 nm and were found to uniformly cover the microfibrous substrates. By pulsed laser deposition-decorating the SWCNHs-coated electrodes with Pt nanoparticles, they were also shown to act as highly effective electrodes for either O_2_ reduction or methanol oxidation, two electrochemical reactions that are crucial to fuel cell technology.

#### 3.1.2. Deposition of Alloy Metal on SWCNHs

SWCNHs were also loaded with alloy-metal catalyst used in fuel cells. 2.5 nm PtRu nanoparticles anchored on both SWCNHs and commercial carbon black were obtained by employing ethylene glycol as the reducing agent [38]. The use of SWCNHs showed catalytic activities 60% higher than using carbon black as the electrocatalyst support in H_2_-fed PEMFC and direct methanol fuel cells (DMFC). In another report, 1.9 nm PtRu nanoparticles assembled with nitrogen-doped carbon nanohorns (NSWCNHs) as an anode catalyst (PtRu/NSWCNHs) exhibited an obvious enhancement in the tolerance to carbonaceous intermediates and the electocatalytic activity for methanol oxidation reaction in comparison to a commercial PtRu/C-JM catalyst and a home-made PtRu/Vulcan catalyst [39].

**Figure 2:**
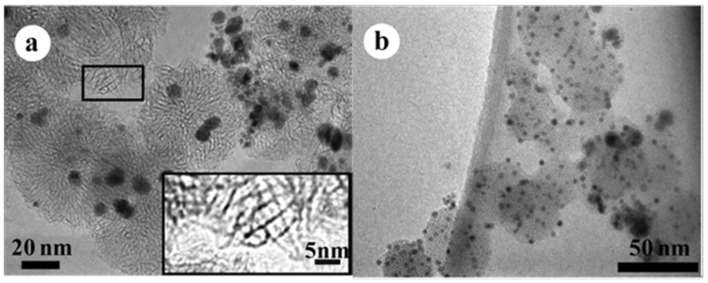
TEM images of (**a**) Pd-Pt/SWCNHs and (**b**) Pd-Au/SWCNHs synthesized by the GI-AIW method. The inset (in Figure 2a) resolves the horn structures of SWCNHs. Reproduced with permission of [40]; American Chemical Society, 2014.

Besides PtRu, SWCNHs bearing Pd alloy nanoparticles that were supplemented with one of nine elements Au, Pt, Cu, Fe, Ni, Ti, Mo, W, and Nb synthesized by a modified gas-injected arc-in-water (GI-AIW) method were investigated [40]. The synthetic method uses a hollow graphite anode into which wires of Pd and an alloying component were inserted to generate arc discharge. PdAu nanoparticles showed significantly high dispersion. The order of average diameter of the nanoparticles as per the alloying component was Au < Pt < Fe < Mo < Ti < Ni < Cu. The tendency of the average size of the alloy nanoparticles was correlated with the ratio of boiling point to melting point, surface tension, and gas diffusivity of the alloying components (Figure 2).

Brandão *et al*. prepared the platinum-free electrocatalysts RuSe deposited on SWCNHs and carbon black as oxygen reduction reaction electrocatalysts’ supports [41]. They studied the tolerance of SWCNHs toward strong catalyzed corrosion conditions and found that SWCNHs have higher electrochemical surface area loss than carbon black or Pt commercial electrodes.

#### 3.1.3. Heteroatom-Doped SWCNHs as Catalyst

**Figure 3:**
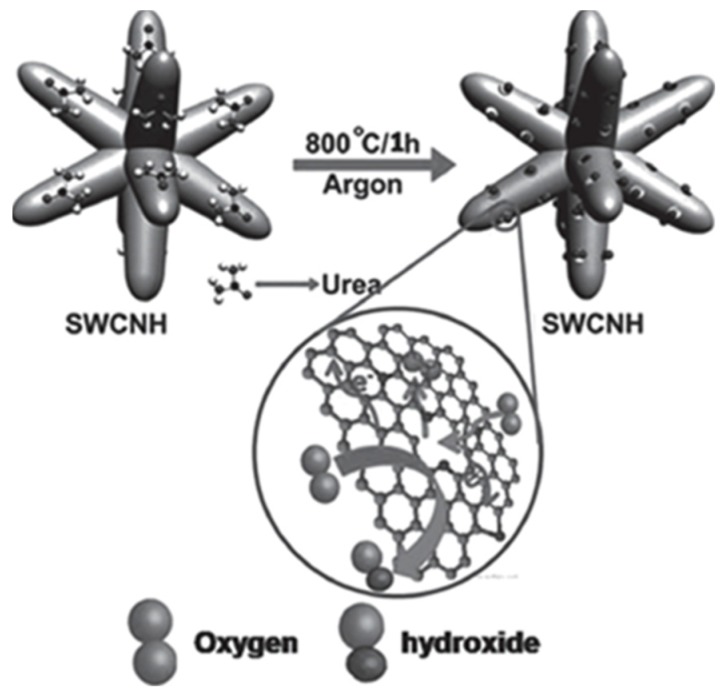
A schematic representation of the synthesis of nitrogen-doped SWCNH by treatment with urea at high temperature. The inset shows the active centers formed around the pore openings, which facilitates the oxygen-reduction reaction. Reproduced with permission of [43]; John Wiley and Sons, 2015.

N-doped SWCNHs have been proven to be better alternatives to Pt in PEMFC [42]. Treatment of SWCNHs with urea at 800 °C produced *N*-doped SWCNHs (Figure 3) [43]. A high surface area of 1836 m^2^·g^−1^ was obtained along with an increased electron conductivity and a high oxygen reduction activity. The above catalyst showed a clear 4-electron reduction pathway at only 50 mV overpotential and 16 mV negative shift in the half-wave potential for oxygen reduction compared to Pt/C along with a high fuel selectivity and electrochemical stability. A membrane electrode assembly (MEA) based on *N*-doped SWCNHs provided a maximum power density of 30 mW·cm^−2^ under anion-exchange membrane fuel cell (AEMFC) testing conditions. The catalytic activities of N-doped SWCNHs were further studied in another report [44]. A simple surface modification of SWCNHs by simultaneous doping with Fe and N at 900 °C (FeNCNH-900) was obtained. Compared to Pt/C, FeNCNH-900 gave a 30 mV improvement in onset potential and a 20 mV gain in half-wave potential in oxygen reduction. Its oxygen reduction reaction activity was still increasing after 1000 cycles. Single-cell fuel cell performance using FeNCNH-900 as cathode catalyst showed a maximum power density of 35 mW·cm^−2^ under alkaline conditions.

### 3.2. SWCNHs for Solar Cells

The development of environmentally friendly, renewable energy is now one of the most important challenges for society. One such type of renewable energy is solar energy. This motivates researchers to develop new technologies and devices that directly convert daylight into electricity efficiently [23,45]. Dye-sensitized solar cells (DSCs) are considered a promising alternative for conventional photovoltaic devices owing to the potentially low production costs, versatility and high efficiency for energy conversion. In general, a DSC consists of three main components: (i) photoelectrode, a porous nanocrystalline TiO_2_ film coated with a monolayer of a dye and deposited onto a transparent conductive oxide (TCO)-coated glass substrate, (ii) an iodide/triiodide redox couple-based electrolyte and (iii) a TCO-coated glass substrate covered by a catalytic material acting as a counter electrode (CE) [46]. Scientists study DSCs in three major areas above mentioned.

**Figure 4:**
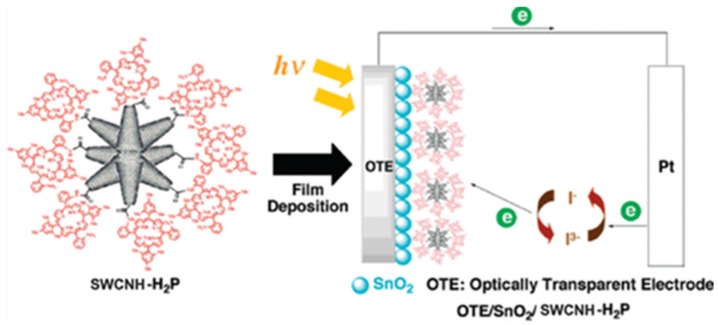
Schematic illustrations of SWCNH-H_2_P structure and a photoelectrochemical solar cell of OTE/SnO_2_/SWCNH-H_2_P. Reproduced with permission of [47]; American Chemical Society, 2008.

Hasobe *et al*. focused on the effect of dyes on the photo-electrochemical solar cell (Figure 4) [47]. They synthesized porphyrin functionalized SWCNHs (SWCNHs-H_2_P) which were implemented into optically transparent electrodes (OTE) cast by nanostructured SnO_2_ films (OTE/SnO_2_) by electrophoretic deposition. The SWCNHs-H_2_P/SnO_2_/OTE electrode displayed an incident photon to current conversion efficiency (IPCE) of 5.8% at an applied bias of 0.2 V *vs.* SCE in a standard three compartment electrochemical cell. The IPCE value was higher than the sum of each component. They found photo-induced electron transfer from the singlet excited state of porphyrin to the nanohorns, while direct electron injection from the reduced nanohorns to the conduction band of the SnO_2_ electrode takes place. Therefore, these processes ensure the generation of photocurrent. Then, they reported a SWCNHs-Zn porphyrin supramolecular assembly (SWCNHs-ZnP) for photo-induced electron-transfer processes [48]. An ammonium cation was attached to SWCNH through a spacer (sp) (SWCNH-sp-NH_3_^+^). Then, SWCNHs-ZnP nanohybrids were assembled by simply mixing crown ether functionalized zinc porphyrin complex (Crown-ZnP) and SWCNH-sp-NH_3_^+^ in DMF. The nanohybrids were then employed to fabricate films onto an OTE/SnO_2_ electrode by drop-cast method. The OTE/SnO_2_/SWCNH-sp-NH_3_^+^-Crown-ZnP electrode exhibited a maximum IPCE of 9% which was a little better than before [47]. Further, they synthesized a dimeric porphyrin [(H_2_P)_2_], which was then grafted onto the SWCNHs to form SWCNH-(H_2_P)_2_ hybrid material [49]. This material was electrochemically deposited onto an OTE/SnO_2_ to produce a photoactive electrode. The maximal IPCE value of 9.6% at 430 nm was observed, which is better than 3.9% for OTE/SnO_2_/(H_2_P)_2_ at 430 nm. The high loading of the electron donor component conjugated onto SWCNHs and its high molar absorptivity are key factors in enhancing and improving photoinduced electron transfer efficiency in SWCNH-based hybrid materials.

**Figure 5:**
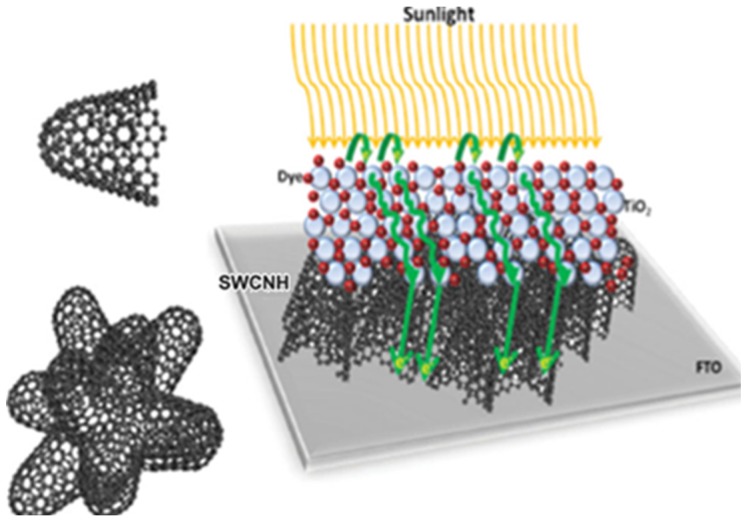
Left: Representation of a SWCNH (**top**) and a bundle-like SWCNH-aggregate present in solution (**bottom**). Right: Schematic representation of the proposed mechanism in the electrode architecture when SWCNH bulk material is implemented as an interlayer between the FTO/TiO_2_. Reproduced with permission of [51]; John Wiley and Sons, 2014.

Guldi *et al*. paid attention to the performance of TiO_2_ electrodes. They explored different nanocarbons (single-walled carbon nanotubes (SWCNTs), graphene, SWCNHs) and their respective oxidized products for doped TiO_2_ photoelectrodes for DSCs and found that all the nanocarbons considerably enhanced the device characteristics and photoresponse as compared with standard TiO_2_ electrodes [50]. SWCNH derivatives are also a valuable dopant for fabricating highly efficient DSCs. Then, they developed a simple spin-coating method to substitute TiCl_4_ with SWCNHs to produce interlayers with controlled thickness (Figure 5) [51]. SWCNHs placed in between fluorine-doped tin oxide and TiO_2_ provides the same or better performance than using TiCl_4_ pretreatment. This circumvents the drawbacks induced by TiCl_4_ such as degradation, acidic nature of TiCl_4_ and waste production. It provides a clean, easy, and eco-friendly alternative to achieve highly efficient cells. Recently, they implemented SWCNHs into ionic liquids based electrolytes for highly efficient solid-state and quasi-solid-state DSCs for the first time [52]. The attachment of SWCNHs to ionic liquids enhanced the miscibility with organic solvent and circumvents the low conductivity of ionic liquids, which was crucial for the overall efficiency of DSCs. Thereby, solid-state DSCs with SWCNHs exhibited rather good performance of 2.09%, which was improved from 0.42% when without SWCNHs. As such, SWCNHs inside solid-state electrolytes enhanced short-circuit current densities (*J*_SC_), whereas open-circuit voltage (*V*_OC_) and fill factors (FF) remained almost unchanged. This was due to a better ionic diffusivity in the electrolyte as well as to a more effective catalytic reduction of I_3_^−^ by SWCNHs. They used 1-butyl-3-methylimidazolium tetrafluoroborate ([BMIM][BF_4_]), 4-tert-butylpyridine (TBP), and guanidinium thiocyanate (GuSCN) as additives to get efficiencies of 7.84% with SWCNHs and only 0.61% without SWCNHs.

The role of SWCNHs is developed further. In 2013, Adélio Mendes’ research group employed SWCNHs as counter electrodes (CE) of DSCs to study the iodide/triiodide redox reaction [46]. By using a half-cell configuration, the high surface SWCNH obtained by partial oxidation of SWCNH mixed with 10 wt % of hydroxyethyl cellulose (HEC) annealed at 180 °C displayed the highest electrocatalytic activity, but a very thick film was needed to perform comparably to a Pt CE. Annealing of such CE at above 400 °C plagued the catalytic activity, in contrast to other studied carbonaceous CEs. Also, redox catalytic activity of SWCNH and HS-SWNH decorated with Pt had the highest electrocatalytic activity per mass of Pt, just requiring 50% of Pt loading to yield the same effect of DSC equipped with a Pt CE. However, such Pt/SWCNH/HEC CE showed half of the transparency.

### 3.3. SWCNHs for Biofuel Cells

**Figure 6:**
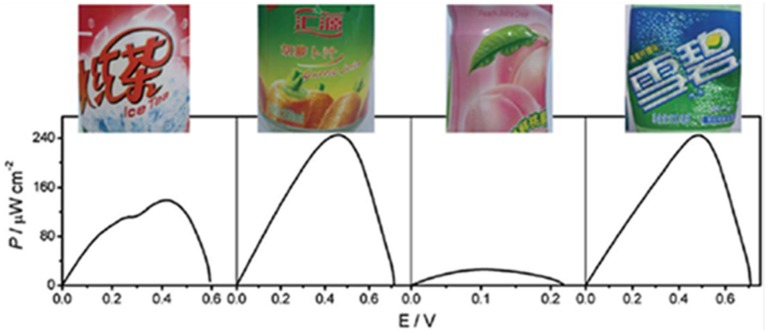
The power outputs of the glucose/air BFC harvesting energy from different kinds of soft drinks (from left to right: iced red tea, vegetable juice, fruit juice, and aerated water). Reproduced with permission of [54]; Royal Society of Chemistry, 2011.

SWNHs possess excellent catalytic properties, high purity, and low toxicities, which make them suitable for bioelectrochemical application. A biofuel cell anode has been developed by using SWCNHs as the support for redox mediators and biocatalysts for the first time [53]. SWCNHs promoted the electropolymerization of methylene blue (MB) and the resulted nanocomposite (poly MB-SWNHs) exhibited prominent catalytic ability for the oxidation of nicotinamide adenine dinucleotide. Glucose dehydrogenase (GDH) was then immobilized on the poly MB-SWNHs modified electrode for the oxidation of glucose. The as-assembled glucose/O_2_ biofuel cell operated at the physiological condition with good performance. A miniature biofuel cell with SWCNHs-modified carbon fiber microelectrodes (CFMEs) as the substrate was reported by the same group [54]. The bioanode was constructed by using GDH as the biocatalyst on SWCNH-modified CFMEs, where a highly efficient and stably confined electrocatalyst for the oxidation of the NADH co-factor of GDH was beforehand immobilized. Similarly, an electrically contacted bilirubin oxidase (BOD)-SWCNHs/CFME was prepared as the biocathode, which exhibited direct bioelectrocatalytic functions for the reduction of O_2_ to H_2_O. The maximum power output of the cell was 140 μW·cm^−2^ at 0.51 V. Most interestingly, the glucose/air biofuel cell can directly harvest energy from different kinds of soft drinks, which could promise potential applications of biofuel cells as portable power sources (Figure 6).

### 3.4. SWCNHs for Solar Thermal Collectors

The use of nanofluids to directly absorb sunlight has been developed in solar thermal collectors recently. In view of better dispersion of SWCNHs in liquid media, much longer time stability of their suspensions, and negligible cytotoxicity, SWCNHs-based nanofluids have been studied by Sani *et al*. [55,56,57]. The thermal conductivity of 0.1 g L^−1^ SWCNHs-H_2_O nanofluids increased with respect to water up to 10%. The light extinction level of SWCNHs-H_2_O nanofluids also increased significantly even at very low concentrations [55]. Compared to Indian inks (about 10% to 16% scattering), SWCNHs have a very low scattering albedo, lower than 5% for red and NIR wavelengths [56], and therefore the absorption effect was strongly prevailing [57]. In addition, the scattering behavior of SWCNHs is independent of the nanohorn morphology (dahlia-like or bud-like) [56]. Further research disclosed that water could be replaced by glycols or water/glycol mixtures to protect against freeze damage and/or to increase the temperature for high temperature solar collectors. Moreover, SWCNHs-ethylene glycol suspension possesses longer time stability and lower ability to agglomerate than amorphous carbon-black particles-ethylene glycol suspensions [58]. Recently, they found that the overall sunlight absorption properties of SWCNHs-based nanofluids could be improved by silver nanoparticles which have good thermal properties [59]. This opens a new interesting route for using such mixed nanofluids as solar absorbers and heat transfer media in solar thermal collectors. A three-dimensional numerical simulation of the nanofluid-based solar receiver using the commercial CFD software FLUENT™ combined with a user defined function (UDF) demonstrated that the use of nanohorn-based nanofluids give rise to a temperature distribution inside the fluid which has its maximum inside the fluid itself, making such nanofluid-based solar collectors quite competitive compared to traditional collectors employing black surface tubes, where the maximum temperature is always reached at the surface [60]. It suggests that SWCNH-based nanofluids are attractive as direct absorbers in solar collectors [57].

## 4. SWCNHs for Energy Storage

The capacitive behavior of SWCNH has shown great potential for application in electrochemical energy storage devices such as supercapacitors and rechargeable batteries.

### 4.1. SWCNHs for Li-Ion Batteries

**Figure 7:**
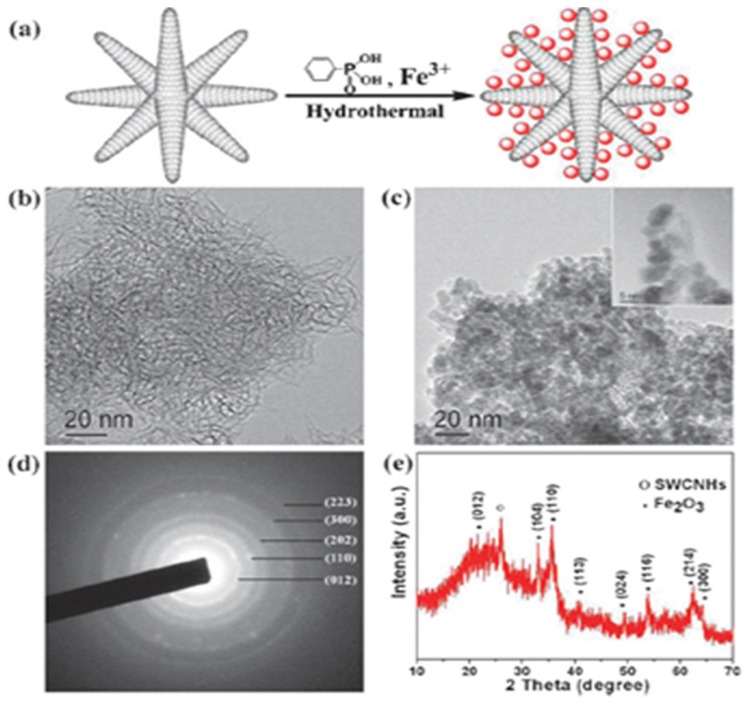
(**a**) Schematic synthesis of Fe_2_O_3_/SWCNHs composite; (**b**) TEM image of SWCNHs. (**c**) TEM image of Fe_2_O_3_/SWCNHs composite inset shows the HRTEM picture of Fe_2_O_3_ particles decorated on the wall of SWCNHs; (**d**) and (**e**) are the corresponding selected area electron diffraction and XRD pattern of Fe_2_O_3_/SWCNHs composite. Reproduced with permission of [62]; Royal Society of Chemistry, 2011.

SWCNHs-metal oxides composite have been applied as anode materials in Li-ion rechargeable batteries. Guan *et al.* developed SnO_2_/SWCNHs and Fe_2_O_3_/SWCNHs composites via a wet chemical method and a hydrothermal method respectively [61,62]. SnO_2_ (2–3 nm) or Fe_2_O_3_ (5*–*8 nm) nanoparticles were homogeneously distributed on the surface of spherical SWCNHs (Figure 7). As anode materials for Li-ion batteries, they showed excellent rate performance and cycle stability. SnO_2_/SWCNHs composite delivered a high capacity of 530 mAh·g^−1^ even after 180 cycles under a current density of 500 mA·g^−1^. As for Fe_2_O_3_/SWCNHs, the reversible specific capacity was stabilized around 550 mAh·g^−1^ even at a high current density of 1000 mA·g^−1^ after 100 cycles. They were much better than most SnO_2_ or Fe_2_O_3_ composites. SWCNHs coated with MnO_2_ nanoflakes have been synthesized via a facile solution method [63]. MnO_2_/SWCNHs composite displayed an excellent capacity of 565 mAh·g^−1^ measured at a high current density of 450 mA·g^−1^ after 60 cylces as anodes for Li-ion batteries. Xu *et al*. prepared nanoporous TiO_2_/SWCNHs composite materials with a simple wet chemistry method and found that the composite exhibited excellent cycling performance and high rate capability as anode materials [64]. The specific charge capacity of the TiO_2_/SWCNHs composite was 100 mAh·g^−1^ at a high rate of 30 °C, which was four times higher than that of pure TiO_2_ anode materials.

Without metal oxides, the mixture of graphite, vapor-grown carbon fibers (VGCFs), and SWCNHs was investigated as anode material for Li-ion batteries [65]. The mixture was heat-treated in Ar atmosphere and carbon-coated by using a chemical vapor deposition (CVD) method (C-graphite/VGCF/SWCNH). The C-rate properties of half-cell for C-graphite/VGCF/SWCNH were superior to those for the mixture of graphite, VGCF, and SWCNHs (C-graphite/VGCF/SWCNH (discharge):3C/0.1C, 85%, graphite/VGCF/SWCNH (discharge):3C/0.1C, 50%), accelerating a promising application for quick charge–discharge of Li-ion batteries.

### 4.2. SWCNHs for Li-S Batteries

The highest energy storage that can be delivered from Li-ion batteries with the abovementioned improvements is still too low to meet the demand of transport or large-scale storage. Li-sulphur (Li-S) batteries using Li metal as the anode, an organic liquid electrolyte, and sulphur composite as the cathode could have very high theoretical capacity (1675 mAh·g^−1^) and specific energy (2567 Wh·kg^−1^) [22]. Guan *et al.* synthesized a novel SWCNH-S composite with high S content up to 76% via a straightforward melt-infusion strategy [66]. The composite exhibited excellent electrochemical performance with a high capacity of 693 mAh·g^−1^ retained after 100 cycles at a high rate of 1.6 A·g^−1^.

### 4.3. SWCNHs for Supercapacitors

Supercapacitors are very attractive due to their high power density and long cycle-life. They could be applied in many fields such as hybrid electric vehicles, portable devices and other renewable energy storage applications [67]. The use of supercapacitors could decrease oil consumption, reduce dependence on oil imports, and effectively solve the problem of urban pollution and pollution from the lead acid battery.

#### 4.3.1. Oxidized SWCNHs for Supercapacitors

**Figure 8:**
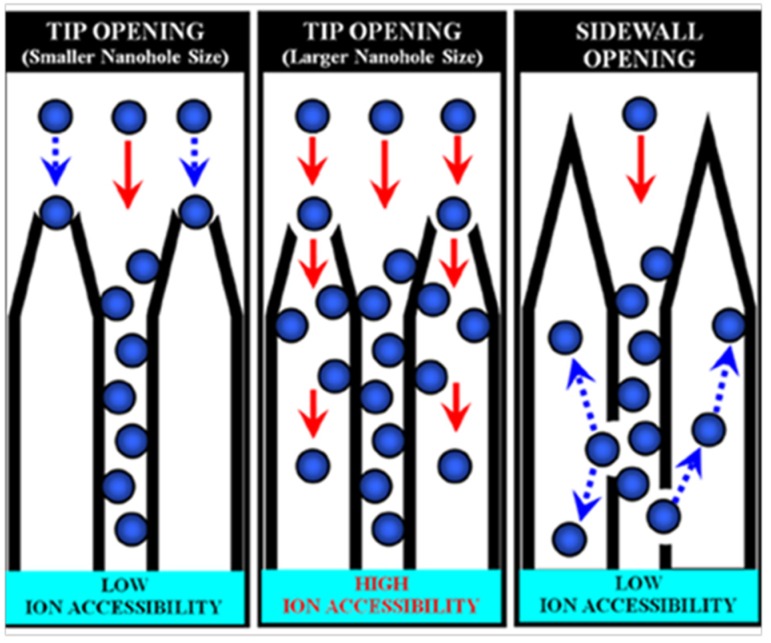
Schematic illustration of ion accessibility with regard to the internal spaces of SWCNHs. Reproduced with permission of [75]; American Chemical Society, 2015.

In 2007, Yang *et al.* applied SWCNHs to fabricate a supercapacitor electrode for the first time [68]. The hole of SWCNHs was opened in O_2_ atmosphere that remarkably increased the micropore surface area and micropore volume. An average internal pore size of 2.4 nm was achieved and was in good agreement with the diameters of individual nanohorns (about 2–3 nm). For aqueous electrolyte of H_2_SO_4_/H_2_O, the specific capacitance of oxidized SWNHs dramatically increased to 114 F·g^−1^ over the unoxidized SWCNHs electrodes (66 F·g^−1^). The enhancement of capacitance might be imparted by a high accessibility of the solvated ions (SO_4_^2^-(H_2_O)*_n_*) to internal pores through the nanowindows. Their study revealed the nanowindow size of SWCNHs is an important parameter for improving the performance of supercapacitors. On the basis of results by Yang’s group, Yuge *et al*. reported the SWCNHs with higher specific surface area as electrodes for capacitor [69]. The maximum value of 1720 m^2^·g^−1^ of oxidized SWCNHs was achieved by heating to 500 °C in air, which is about 20% greater than that of holey nanohorns previously reported [70,71]. However, further increase of temperature leads to lowered specific surface area, which might be aroused by excessive oxidation that removed pore walls of SWCNHs and left the graphene-sheet structure of high stability against combustion in O_2_. Oxidized SWCNHs displayed linear voltage-time dependence and specific capacitance of 100 F·g^−1^. This implies that the specific capacitance increases with the surface area, in accordance with the fact that the nano-sized inner space of a cylindrical structure such as nanotubes and nanohorns permits charge storage [72,73]. Yang *et al*. systematically studied the relationship of the specific effective surface area and electrical conductivity of SWCNHs and accessibility of the electrolyte ions in the SWCNH-based supercapacitor [74]. Heat treatment of SWCNHs leads to an increased ratio of sp^2^/sp^3^ hybridized carbon, which improves the electrical conductivity of SWCNHs. In spite of the slightly reduced specific surface area, as a result of heat treatment, the specific capacitance per specific surface area of the SWCNH electrode remarkably increased from 22 to 47 μF·cm^−2^. Such a result clearly demonstrates an explicit enhancement in accessible effective surface area by electrolyte ions. Therefore, a high degree of utilization for the interstitial pore of SWCNHs by solvated ions is pivotal in achieving high volumetric capacitance of SWCNH-based supercapacitors. They further found the size and position of nanoholes with regard to ion accessibility are crucial factors to improve the capacitive performance of SWCNH-based supercapacitors using an ionic liquid electrolyte [75]. The oxidized SWCNHs at 673 K showed a low specific capacitance per unit of internal specific surface area (4.0 μF·cm^−2^), as the nanoholes created on the tips of SWCNHs via a selective chemical attack are too small to introduce electrolyte ions. For a sample oxidized at 723 K, the enlarged diameter of the nanoholes on the tips allows electrolyte ions to penetrate into the internal spaces of the SWCNHs, leading to a 2-fold capacitance improvement (8.6 μF·cm^−2^). Further increasing the temperature to 823 K destroyed the capacitance, which can be tentatively attributed to the selective formation of nanoholes on the sidewalls of the SWCNHs, where the small interstitial pores restrict ion diffusion to deeply positioned nanoholes on the sidewalls of the SWCNHs (Figure 8).

#### 4.3.2. SWCNH-Polymer Composite for Supercapacitors

Wei’s group reported the first simple template-free electrochemical galvanostatic synthesis of polyaniline (PANI) nanobrushes and PANI-SWCNH hybrid material [76]. When the ITO surface was coated with PANI nanobrushes, the capacitive current increased, which was attributed to the pseudocapacitance current. The specific capacity is about 48 F·g^−1^. When PANI-SWCNH hybrids were used as electrodes in supercapacitors, the capacitive current improved considerably with a specific capacity of 168 F·g^−1^. Khatua also reported the in situ polymerization of aniline in the presence of SWCNH and acidic medium (HCl) [77]. The composites showed high electrical conductivity in the order of 6.7 × 10^−2^ S cm^−1^ which indicates the formation of a continuous interconnected conducting network path in the polyaniline/carbon nanohorn (PACN) composites. Thus, the afforded fiber-like composites displayed high specific capacitance value of ca. 834 F·g^−1^ at 5 mV·s^−1^ scan rate compared to 231 F·g^−1^ for pure PANI and 145 F·g^−1^ for SWCNH under the same conditions.

#### 4.3.3. SWCNH Composites with Other Nanocarbons for Supercapacitors

##### 4.3.3.1. SWCNH-SWCNT

Hiralal *et al.* explored the application of SWCNHs and SWCNTs in thin film supercapacitors [78]. SWCNT films were deposited onto polyethylene terephthalate (PET) by vacuum filtration methods, onto which SWCNH were deposited by a drop-cast method in water. The combination of SWCNH and SWCNT renders an enhanced specific capacitance which stems from the high porosity (75%) of SWCNH and high permeability (5100 mDarcy). They demonstrate the possibilities that may be available for the enhancement of electrodes by tailoring and combining relevant materials hierarchically in multiple scales. Hata et al. also prepared an electrode with SWCNTs (20 wt %) and SWCNHs (80 wt %) where the SWCNT act as the framework [79]. The electrode in supercapacitor exhibits a high maximum power rating (990 kW·kg^−1^; 396 kW·L^−1^) surpassing other electrodes. Despite the low surface area (280 m^2^·g^−1^ from nitrogen adsorption) of the electrode, the larger meso-macro pore volume (2.6 *vs.* 1.6 mL g^−1^ from mercury porosimetry) benefits the retaining of more electrolyte in electrode, ensuring facile ion transport. Owing to the monolithic chemical composition and mechanical stability, the novel composite electrode also exhibited durable operation, 6.5% decline in capacitance over 100,000 cycles being accomplished.

##### 4.3.3.2. SWCNH-Graphene

**Figure 9:**
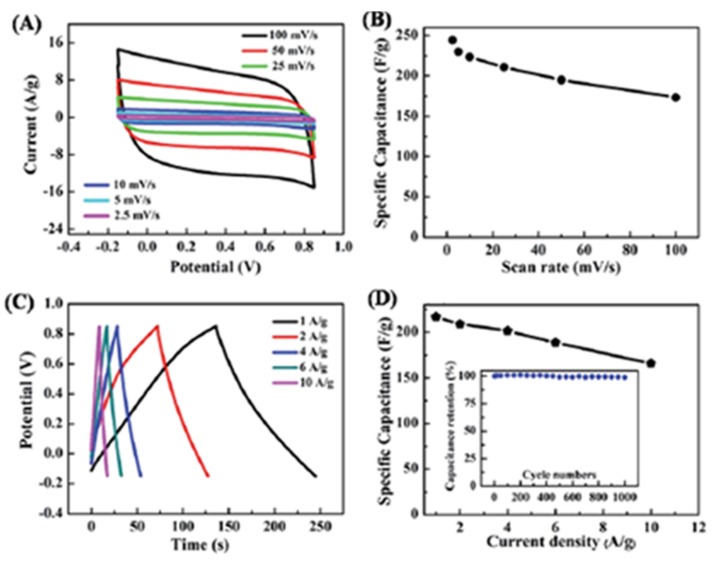
Electrochemical characterization of G/SWCNHs in 1 M KOH aqueous solution at room temperature. (**a**) CV curves at different scan rates. (**b**) Specific capacitance *versus* scan rate. (**c**) GCD curves at different current densities ranging from 1 A·g^−1^ to 10 A·g^−1^. (**d**) Specific capacitance as a function of current density; inset shows capacitance retention *versus* cyclic number. Reproduced with permission of [81]. Royal Society of Chemistry, 2015.

Khatua *et al*. explored the performance of SWCNH-graphene-hybrid-based electrodes in capacitors [80]. The hybrid was prepared by simply mixing, ultrasonication and agitation of SWCNH, graphene and Cetyl trimethylammonium bromide (CTAB). A specific capacitance of 677 F·g^−1^ at a scan rate of 5 mV·s^−1^ is achieved. They attributed this to the several points below. First, there is a strong π-π stacking interaction between the conducting graphene nanoplate (GNP) and SWCNH. Second, GNPs are well and homogeneously coated by SWCNH in the composite, which facilitates easy ion transfer through the hybrid and reduces the ionic diffusion route. Finally, a strong interconnecting conducting network might be developed by the conducting SWCNH and GNP, which is also responsible for the high capacitance of the hybrids. Stability testing shows that 81% of specific capacitance retained after 1000 cycles in 1 M KCl electrolyte at a scan rate of 10 mV·s^−1^. Tao’s group for the first time reported an environmentally friendly hydrothermal synthesis of heterostructure of nanoporous graphene/SWCNHs (G/SWCNHs) hybrid [81]. Graphene in the hybrid acts as scaffolding, while the SWCNHs prevent the graphene nanosheets from stacking face-to-face. The xerogel-like G/SWCNHs hybrids have ultra-micropores of *ca.* 0.6 nm and mesopores of 2*–*12 nm with the total nanopore volume of 0.20 cm^3^·g^−1^. An enhanced capacitance of 244 F·g^−1^ in 1 M KOH with ultra-fast charge-discharge and excellent rate capability is achieved, superior to reduced graphene oxide, SWCNHs and SWCNH composites. The hybrid is promising for practical high power density applications due to a capacitance retention of 99% after 1000 cycles at a current density of 10 A·g^−1^ (Figure 9).

#### 4.3.4. SWCNHs-Metallic Oxide Composite for Supercapacitors

In 2011, Guan *et al.* prepared a ternary thin film electrode created by coating MnO_2_ onto a network composed of SWCNTs and SWCNHs [67]. As the scaffold, SWCNTs confer good conductivity, mechanical stability and high surface area for the ternary nanocomposites electrode. SWNHs play a key role in tuning the pore structure of the electrode, facilitating the rapid transport of the electrolyte ions and increasing the electrochemical utilization of MnO_2_ pseudocapacity; the MnO_2_ layer was directly coated on carbon matrix, providing the main pseudocapacitance and enabling fast electron transfer within electrodes. These features allow a higher rate of electrolyte infiltration, facilitate ion insertion/extraction and electrons transport in the electrode and thus decrease the ion diffusion path and electron transport resistance. Contributing from all these factors, the maximum specific capacitances of 357 F·g^−1^ for this ternary nanocomposites electrode at 1 A·g^−1^ were achieved in 0.1 M Na_2_SO_4_ aqueous solution. Further, the ternary nanocomposite electrode shows an excellent rate capability with specific capacitance preserved 65% with current density increasing from 1 to 30 A·g^−1^ and good cycling performance of 98.5% retention after 2000 cycles. In 2013, Shelke *et al*. studied the application of Fe_3_O_4_-SWCNH nanocomposite for supercapacitor electrodes [82]. The surface area of Fe_3_O_4_-SWCNH increases to 655 m^2^·g^−1^ as compared to pristine SWCNH (392 m^2^·g^−1^). The specific capacitance of Fe_3_O_4_-SWCNH electrodes reach up to 377 F·g^−1^. The high specific capacitance of the composite electrode is due to the high surface area, high electrical conductivity and complimentary morphology of the SWCNHs in the nanocomposite, which decrease the charge transfer resistance of Fe_3_O_4_. Meanwhile, the capacitor based on the composite electrode has an excellent life-cycle. The composite electrode supercapacitors retain 93% of their initial capacitance after 1000 cycles.

### 4.4. SWCNHs for Hydrogen Storage

Hydrogen is a flexible energy carrier that can be produced from any regionally prevalent primary energy source. It can be effectively transformed into any form of energy for diverse end-use applications. Moreover, hydrogen is particularly well suited for use in fuel cells that efficiently use hydrogen to generate electricity. Hydrogen, with its low-carbon footprint, has the potential to lower local air pollutants and noise emissions compared to direct fossil fuel combustion, and facilitate significant reductions in energy-related CO_2_ emissions and to contribute to limiting global temperature increases. Development of efficient hydrogen storage materials is one of the key tasks in the field of hydrogen energy.

Chen *et al*. investigated hydrogen storage ability of SWCNHs via theoretical calculation [83]. Due to severe curvature, SWCNHs could bind hydrogen molecules through enhanced binding at the top section adjacent to its closed top end. The storage capacity limited by the room at the top end section was only 1.8 wt % for hydrogen to be captured inside the studied nanohorn. Li atoms were found to adhere on the sidewalls of nanohorns separately at low Li atom content rather than aggregate. Each Li atom on the outer sidewall could bind three hydrogen molecules, while the small room inside the nanohorn limited the adsorbed hydrogen molecules to be eight at maximum. By adsorbing 24 more H_2_ with binding energy around 160 meV/H_2_ on the outer sidewall at the large hollow space circled by neighboring Li atoms and their attracted hydrogen molecules, the capacity of 8.6 wt % could be obtained. Pagura *et al*. reported a method for large-scale production of SWCNHs in a prototype reactor with high quality material formation at rates of about 100 g·h^−1^ [84]. The scale can be increased up to ten times, making it possible to really use SWCNHs in hydrogen storage.

Metal nanomaterials could increase hydrogen storage on SWCNHs. Experimental measurements of metal-assisted hydrogen storage have been hampered by inaccurate estimation of atomically stored hydrogen deduced from comparative measurements between metal-decorated and undecorated samples. Liu *et al*. reported a temperature cycling technique combined with inelastic neutron scattering (INS) measurements of quantum rotational transitions of molecular H_2_ to more accurately quantify adsorbed hydrogen aided by catalytic particles using single samples. Temperature cycling measurements on SWCNHs decorated with 2*–*3 nm Pt nanoparticles showed 0.17% mass fraction of metal-assisted hydrogen storage (at approximate to 0.5 MPa) at room temperature. No additional metal-assisted hydrogen storage was observed in SWCNH samples without Pt nanoparticles cycled to room temperature. The possible formation of C-H bonds due to spilled-over atomic hydrogen was also investigated using both INS and density functional theory calculations [85]. Moreover, the amount of H_2_ absorbed by SWCNHs containing Pd-Ni alloy nanoparticles (Pd-Ni/SWCNHs) prepared by the gas-injected arc-in-water method was larger than the predicted combined absorption contributed by Pd-Ni alloy and pure-carbon SWCNHs [86]. This synergetic H_2_ absorption was induced by the combination of Pd-Ni alloy nanoparticles and SWCNHs and occurred because of a spillover effect. Opening the pores of Pd-Ni/SWCNHs by a mild oxidation treatment drastically improved the H_2_ absorption. Consequently, the diffusivity of H_2_ into Pd-Ni/SWCNHs was enhanced to the point where H_2_ absorption could reach saturation in an extremely short time.

## 5. Conclusions

SWCNTs have been a field of active scientific research for the last two decades. Due to its unique electronic, structures and chemical properties, this material has found various applications in contemporary advanced technologies. The physical and chemical properties of SWCNHs can be controlled through heteroatom-doped, molecular-functionalized, noble metal catalysts or other nanocarbon-material-hybrid SWCNHs. Introduction of this kind of material into energy conversion and storage fields opens new perspectives and results in outstanding performances. 

This paper presents an overview of this exciting area. In the field of energy conversion, SWCNHs could be used as catalyst support for single-metal or double-metal nanoparticles, or as catalysts after heteroatom doping for fuel cells, such as ethanol fuel cells. In solar cells, SWCNHs are used as light conversion support and functioned by various dye molecules and metal complexes. Moreover, it was used as an interlayer, as opposed to electrodes or electrolytes implemented by ionic liquids. Also, SWCNHs have been studied in biofuel cells as support, biocatalysts, or electrode substrates. In brief, the role of SWCNHs is diversified. However, the study is still in its infancy. There is a critical need to synthesize novel SWCNH-based materials for energy conversion and to exploit their role. For example, considering lower costs, the development of heteroatom-doped SWCNHs appears to be one of the main driving forces for fuel cell research in the long term. The catalytic ability may be adjusted by controlling doping proportion and the position of the heteroatom. Further functionalization of heteroatom-doped SWCNHs by metal nanoparticles, light conversion molecules and other carbon nanomaterials opens new perspectives on the energy conversion applicability of SWCNHs.

In the field of energy storage, SWCNHs used in Li-ion batteries, Li-S batteries, supercapacitors and hydrogen storage are discussed in detail. Some monometal SWCNHs, alloy SWCNHs, metal oxide SWCNHs, S-SWCNHs, SWCNT-SWCNHs, grapheme SWCNHs, and polymer-SWCNH composites were synthesized and applied. However, this kind of SWCNH-based composite is still limited. The introduction of new organic or inorganic nanomaterials with a specific design is essential to the massive use of SWCNHs in these fields. There is an urgent need to exploit the roles of SWCNH-based composites, not just for use in electrodes, and to enhance their performance, which involves developing high-surface-area SWCNH-based composites with tailored pore sizes and modification of their surface characteristics and nanoarchitecture design.
